# Flow cytometric methodology for the detection of *de novo* human T-cell leukemia virus -1 infection *in vitro*: A tool to study novel infection inhibitors

**DOI:** 10.1016/j.jviromet.2019.113728

**Published:** 2019-12

**Authors:** Carina Peres, Yuetsu Tanaka, Fabiola Martin, James Fox

**Affiliations:** a Department of Biology & Hull York Medical School, University of York, UK; b Graduate School of Medicine, University of the Ryukyus, Okinawa, Japan

**Keywords:** Tax, Flow cytometry, HTLV-1, Human T-lymphotropic virus-1, Human T-cell leukemia virus

## Abstract

Methodology to detect and study *de novo* human T-cell leukemia virus (HTLV)-1 infection is required to further our knowledge of the viruses’ mechanisms of infection and to study potential therapeutic interventions. Whilst methodology currently exists, utilisation of an anti-Tax antibody to detect *de novo* Tax expression in permissive cells labelled with cell tracker allowing for the detection by flow cytometry of new infection after co-culture with donor cell lines productively infected with HTLV-1 is an alternative strategy. Using this methodology, we have been able to detect *de novo* infection of the T cell line HUT78 following co-culture with the productively infected HTLV-1 donor cell line MT-2 and to confirm that infection can be effectively blocked with well characterised infection inhibitors. This methodology will benefit experimental studies examining HTLV infection *in vitro* and may aid identification of therapeutic agents that block this process.

## Highlights

• MT-2 cells express more Tax, gp46 and p19 than HUT102′s.
• HUT78 cells express higher levels of the HTLV-1 permissive receptors neuropilin and GLUT-1 than CEM or JURKAT.
• Irradiation does not eliminate all MT-2 donor cells in HTLV-1 co-culture protocols.
• Flow cytometry and Lt-4 anti-tax antibody can detect *de novo* HTLV-1 infection at early time points.
• Cytochalasin B and sodium valproate inhibit HTLV-1 infection at early time points.

## 1 Introduction

Worldwide an estimated 10–15 million people live with HTLV-1 (Gessain and Cassar, 2012), with areas of high endemicity in Japan, West Africa, South America, Caribbean islands, Romania, Iran, Middle East and Australo-Melanesia, where the prevalence ranges from 3 to 50% (Einsiedel et al., 2016; Gessain and Cassar, 2012; Murphy, 2016). There is currently no cure for HTLV-1 infection or its associated diseases and it has been considered a neglected virus. HTLV-1 is a milk- and blood-borne and sexually transmitted virus causing significant mortality and morbidity in patients who develop disease such as HTLV associated myelopathy/Tropical Spastic Paraparesis (HAM/TSP) and adult T-cell leukemia/lymphoma (ATL), making HTLV-1 a significant threat to public health. Translational research is required to identify pre- and post-exposure interventions similar to HIV-1 infection.

*In vitro* HTLV-1 infection studies and syncytium-assays are used to further our understanding of the mechanisms of HTLV-1 infection and to investigate novel drug inhibitors. *In vitro* cell-free HTLV-1 infection has been reported (Fan et al., 1992; Jones et al., 2008), but is an inefficient process. Therefore co-cultures of productively infected HTLV-1 donor cells with permissive cells are commonly used to study *in vitro de novo* infection (Feuer and Green, 2005). Typically, new infection is detected through polymerase chain reaction of HTLV-1 specific Tax DNA after 24 h of co-culturing permissive cells with irradiated donor cells followed by several cycles of media changes (Balestrieri et al., 2008). However, we monitored MT-2 cell viability after 30 Gy of X-ray irradiation and discovered low numbers of viable cells persist in culture. Therefore, we observe several potential problems with the currently established and widely utilised methodology: irradiation and media changes may not fully remove donor DNA and thus potentially deliver false positive results in future experiments. Also, since sufficient time is required to detect Tax DNA, the study of HTLV-1 cell-to-cell transmission at an early stage remains problematic and, for efficient discovery of pre- and post-exposure prophylaxis interventions, a good understanding of early HTLV-1 infection stage is needed.

Recently, novel methodology utilising reporter systems transfected into permissive cells to drive luciferase expression following HTLV-1 infection has been reported (Gross and Thoma-Kress, 2017). Here, we describe the novel use of labelling and flow cytometry gating strategies to determine early infection stage of *in vitro de novo* HTLV-1 infection, without the need to irradiate or eliminate donor cells. Flow cytometry is a powerful tool with a wide variety of applications that fundamentally interrogates single particles “flowing” through a detector system (Jahan-Tigh et al., 2012). Flow cytometry allows simultaneous multi-parametric measurements of physical and chemical characteristics of thousands of particles per second. Particles tested are commonly cells, which can be labelled with fluorescent dyes or cell specific fluorescent antibodies and we have utilised both these strategies to delineate permissive- from donor-cells to detect HTLV-1 specific proteins indicative of early stage *de novo* infection. In addition, this methodology could be used to identify novel cell-to-cell transmission targets through HTLV-1 infection inhibitors, in more physiological systems such as organo-typical explant models to advance the identification of specific HTLV-1 cell entry inhibitors.

## 2 Methods

### 2.1 Cells and co-cultures

HUT78 cells were from the NIBSC, Potters Bar, UK, donated by Dr A Doyle, ECACC (Gazdar et al., 1980); MT-2 cells (Miyoshi et al., 1981) were a gift from Graham Taylor, Imperial College, London. Both cell lines were routinely cultured under standard tissue culture conditions, 37 °C with 5% CO_2_ in air, in Roswell Park Memorial Institute 1640 (RPMI) containing 10% FBS, 2 mM L-glutamine, 10 mM HEPES, 100 U/ml penicillin and 100 μg/ml streptomycin (all from Invitrogen, Paisley, UK).

Prior to co-culture establishment, HUT78 cells were harvested and resuspended at 1 × 10^6^ cells/ml in pre-warmed serum-free RPMI media containing CellTracker Orange CMRA dye (Invitrogen) used 1:5000 for 15 min at 37 °C. Cells were centrifuged and resuspended at 1 × 10^6^ in serum-free RPMI alone for 30 min at 37 °C. Cells were then centrifuged again and resuspended at 0.8 × 10^6^ cells/ml for co-culture. Co-cultures were established between the HUT78 and MT-2 cell lines at a 1:1 ratio for 24 h at a seeding density of 0.8 × 10^6^ cells/ml; mono-cultures of cells treated in the same way were established at the same cell density to serve as controls. Co-cultures were also established in the presence of 10 μM cytochalasin B (Fisher Scientific, Loughborough, UK) or 0.5 mM sodium valproate (Santa Cruz Biotechnology, Heidelberg, Germany).

### 2.2 Antibodies, labelling and flow cytometry

Following co-culture, cells were collected and fixed in 2% ultrapure methanol free paraformaldehyde (Park Scientific, Northampton, UK) in PBS for 10 min at room temperature, washed and resuspended in permeabilisation buffer: PBS + 7% normal goat serum + 0.2% saponin (Goon et al., 2002) for 10 min. Cells were stained using an optimised concentration of anti-Tax antibody (clone: Lt-4; kindly provided by Tanaka Y, Ryukyu University (Lee et al., 1989)), or equivalent concentration of IgG3 isotype control (eBioscience, Altrincham, UK), in permeabilisation buffer for 20 min. Following two washes in permeabilisation buffer, cells were incubated in permeabilisation buffer containing goat anti-mouse IgG3:SureLight APC conjugated secondary antibody (Cambridge Bioscience, Cambridge, UK) for 20 min. Two further washes with permeabilisation buffer followed by two washes with PBS were performed prior to analysis on a FACSArray flow cytometer (BD Biosciences, Oxford, UK). In the co-cultures, a gating strategy was devised that selected for cells positive for CellTracker Orange in the FSC/SSC region of HUT78 cells. A region gate was established using the isotype controls such that the percentage of cells staining positive with the anti-Tax antibody could be calculated. To examine MT-2 cells and for the monoculture MT-2 control, a gating strategy was established for the cells based upon MT-2 FSC/SSC and events negative for CellTracker Orange.

### 2.3 Control: Tax quantification by real time polymerase chain reaction

DNA was extracted from cell lines using the QIAamp DNA mini kit (Qiagen, Manchester, UK) before being serially diluted to establish standard curves consisting of 20,000 to 2 β-globin copies and 70,000 to 7 HTLV-1 Tax copies, as previously described (Demontis et al., 2013). Real time polymerase chain reaction (PCR) was performed using fast SYBR green mastermix (Applied Biosystems, Loughborough, UK) and 2.5 μM or 5 μM of each forward (F) and reverse (R) primer for Tax (F: 5′-CGGATACCCAGTCTACGTGT-3′, R: 5′-GAGCCGATAACGCGTCCATCG-3′) or β-globin (F: 5′-GCAAGGTGAACGTGGATG-3′, R: 5′-TAAGGGTGGAAAATTGACC-3′), respectively. PCR was performed on an Applied Biosystems Prism 7300; thermal cycler conditions were: 95 °C for 20 s, followed by 40 amplification cycles of 95 °C for 15 s and 60 °C for 60 s. Tax DNA copy number was calculated from the standard curves normalized to the number of β-globin copies.

## 3 Results

### 3.1 Incomplete removal of HTLV-1 donor cells by irradiation

MT-2 cell viability was monitored daily after a relevant irradiation dose (30 Gy); small numbers of cells, on average 0.12 × 10^6^ cells/ml, persisted to 240 h (Supplementary Fig. 1). A higher irradiation dose, 200 Gy, was tested but a small proportion of the cells again remained viable, on average 0.07 × 10^6^ cells/ml (Supplementary Fig. 1). These experiments were replicated by other researchers with similar results (Personal communication, David Brighty, University of Dundee). A second productively infected HTLV-1 cell line, HUT102, was compared and, similarly, a small percentage also survived to 240 h after 30 Gy irradiation (data not shown).

### 3.2 Optimisation of our *in vitro* de novo cell-to-cell infection system

HUT78 cells were selected as our permissive cells; amongst available T cell lines (HUT78, CEM and Jurkat) they expressed greatest neuropilin and GLUT-1 (Supplementary table 1), cell surface mediators of HTLV-1 entry. MT-2 cells were selected as our HTLV-1 donors since they express 1.68 times more Tax by qRTPCR than HUT102 and express markedly higher levels of Tax, p19 and gp46 by flow cytometry (Supplementary Table 2).

### 3.3 Establishment of a flow cytometry system capable of detecting *de novo* HTLV-1 infection in permissive cells after co-culture

HUT78 cells are discernible from MT-2 cells based upon forward and side scatter properties (Supplementary Fig. 2) but we loaded HUT78 cells with a fluorescent cell tracker dye prior to the co-culture experiments to allow more robust flow cytometric delineation. Typically, >95% of HUT78 cells become stained and are strongly, fixation resistant, fluorescent after dye incubation, making them unmistakable from MT-2 cells (Supplementary Fig. 2). As per manufacturer declarations, HUT78 cells were unable to transfer dye to other cells in co-culture when monitored for up to three days (data not shown).

**Fig. 1:**
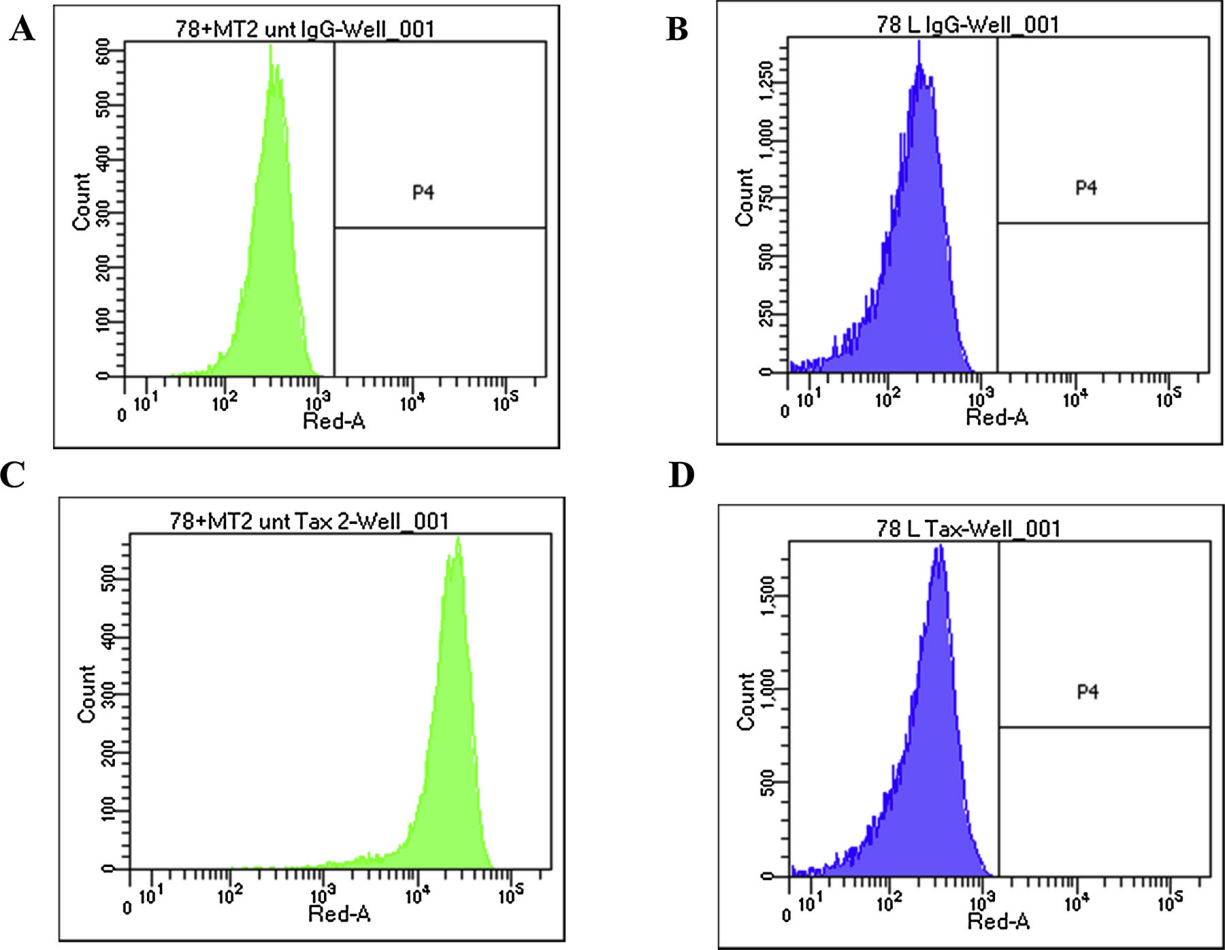
**MT-2 cells show positive Tax expression whilst HUT78 cells are negative.** Representative histograms of three separate experiments performed in triplicate showing averaged data of at least 5000 acquired cells of MT-2 cells (A, C) or HUT78 cells (B, D) stained with isotype control (A, B) or anti-Tax antibody (C, D).

Productively HTLV-1 infected MT-2 cells express high levels of HTLV-1 specific p19, gp46 and Tax; HTLV-1 naïve HUT78 cells are negative for Tax by intracellular flow cytometry (Fig. 1) and immunofluorescence (Supplementary Fig. 3) and lack p19 and gp46 expression (data not shown). Initially, the cell-surface markers p19 and gp46 were trialled as markers of *de novo* HTLV-1 infection of HUT78 cells after co-culture with HTLV-1 donor MT-2 cells and indeed HUT78 appeared to stain positively for these markers after a 24 -h co-culture (data not shown). However, the addition of the protein synthesis inhibitor cycloheximide into the co-culture system did not completely abolish detection of p19 and gp46 expression on co-cultured HUT78, which could not sufficiently rule out potential membrane transfer between MT-2 and HUT78 cells as the source of p19 or gp46 on the HUT78 cells. We therefore chose the expression of intracellular Tax as the marker for *de novo* infection of naive HUT78 cells with HTLV-1, which cannot be attributed to membrane transfer.

### 3.4 Flow cytometric gating strategy

The ability to discern HUT78 cells from MT-2 donor cells forms a critical part of our flow cytometry gating strategy. Cellular debris, commonly observed in long-term cell cultures, are first excluded by forward / side scatter segregation. The cells of interest are enriched by a size exclusion gate, after which any none-fluorescent cells, *i.e.* MT-2 cells, or HUT78 cells that had not taken up cell-tracker stain, are then excluded from future analyses using a conservative segregatory gating strategy on only strongly fluorescent cells (Supplementary Fig. 2).

### 3.5 Analysis of HTLV-1 infection in HUT78 by MT-2 cells at 24 h post co-culture and infection inhibition studies

**Fig. 2:**
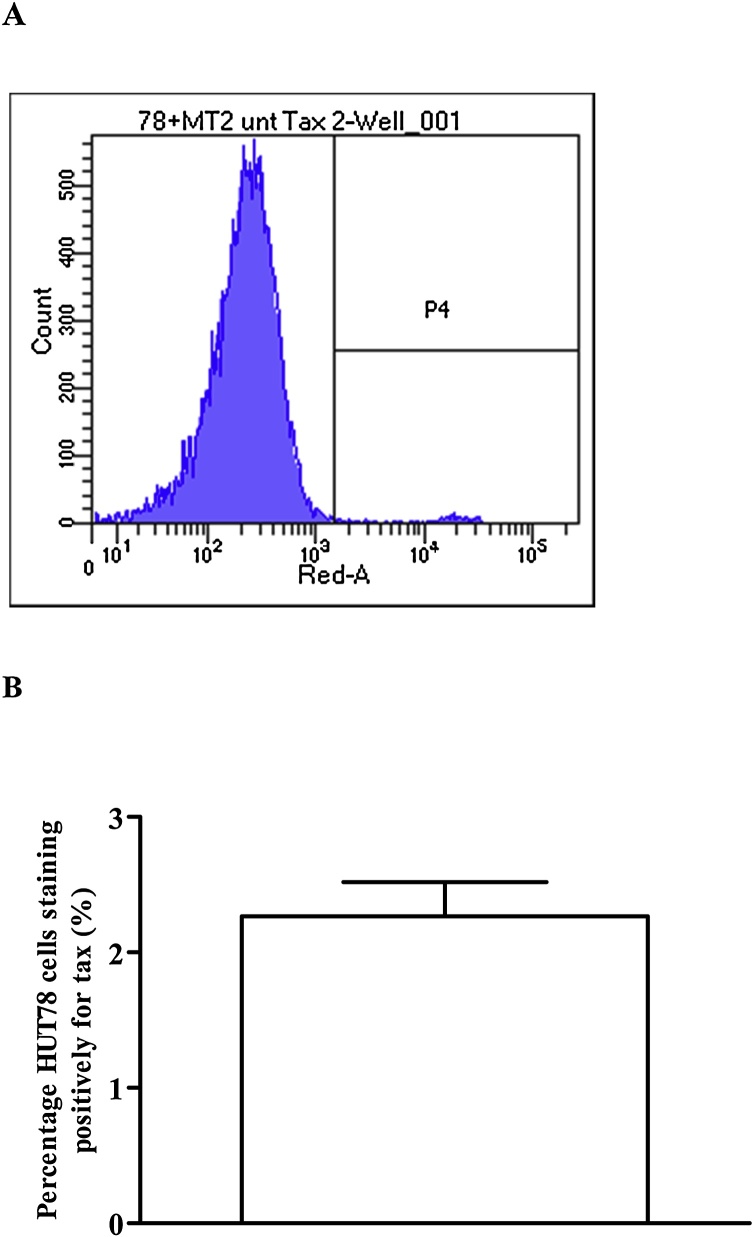
**HUT78 cells become Tax positive after co-culture with MT-2 indicative of *de novo* HTLV-1 infection.** Representative histogram of three experiments performed in triplicate showing averaged data of at least 5000 acquired cells: events in the P4 gate are HUT78 cells staining positive for Tax (A) and average quantified data, mean ± S.D. (B).

When HUT78 cells were co-cultured 1:1 with MT-2 cells, a population of approximately 2.3% expressed Tax (Fig. 2) as visualised through flow cytometry. Hence, *de novo* HTLV-1 infection of HUT78 was detectable in this system at an early time point of only 24 h.

**Fig. 3:**
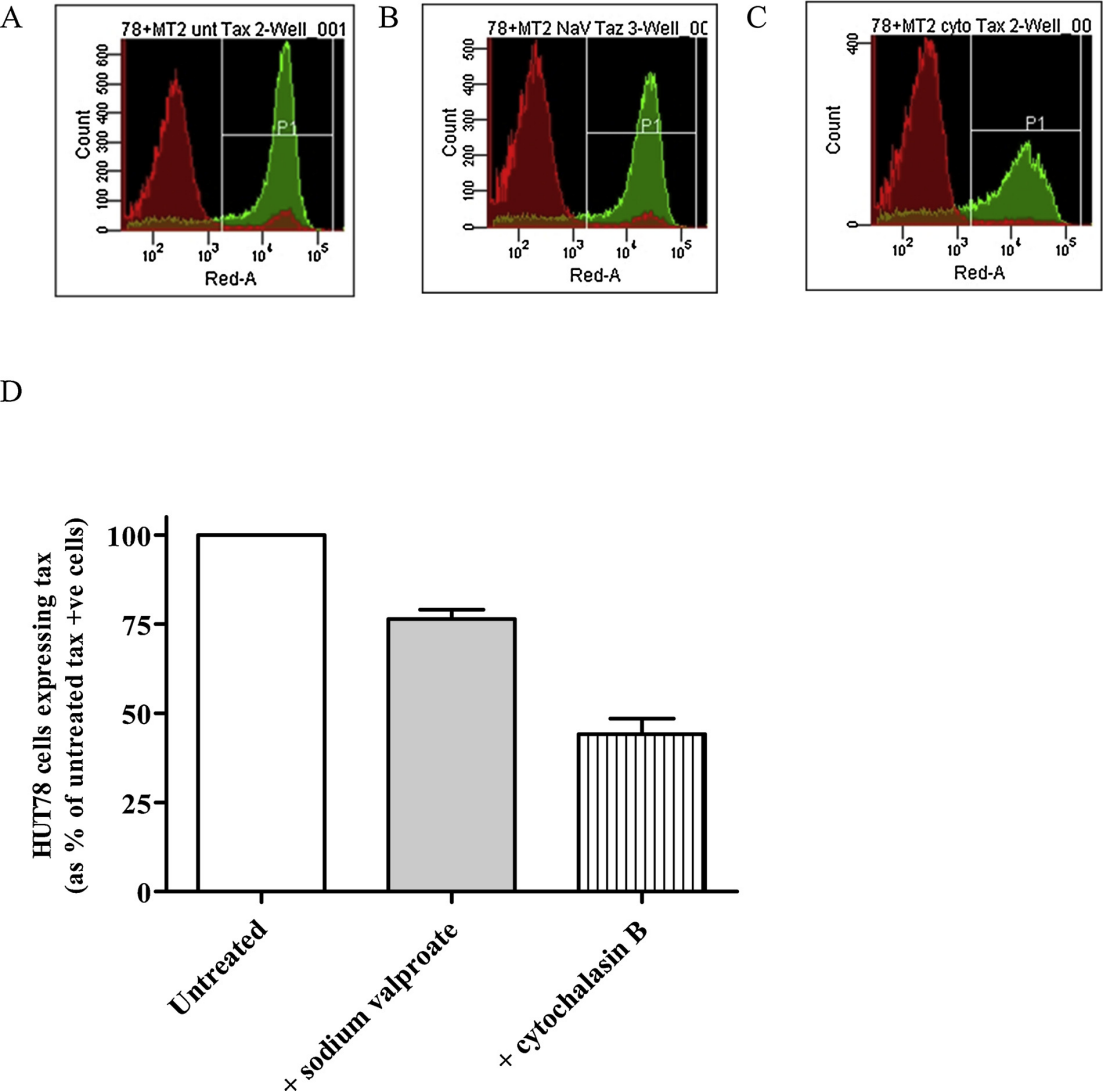
**Sodium valproate and cytochalasin B inhibit*de novo*HTLV-1 infection of HUT78 cells.** HUT78 cells were co-cultured with MT-2 in the absence (A) or presence of sodium valproate (B) or cytochalasin B (C) before flow cytometric staining with an anti-Tax antibody (A–C) and quantification (D) of the percentage of Tax-positive HUT78 cells shown in the histograms within the P1 gate. HUT78 cells are coloured red on the histograms, MT-2 cells are green. Data is a representative experiment performed in triplicate (For interpretation of the references to colour in this figure legend, the reader is referred to the web version of this article).

The HTLV-1 infection inhibitors cytochalasin B (Manel et al., 2003) or sodium valproate (Afonso et al., 2010) were utilised to validate our demonstration of *de novo* infection. We conducted two inhibition studies, firstly the addition of cytochalasin B, which has been shown to inhibit HTLV-1 infection *via* GLUT-1 receptor interaction, rather than impacting actin microfilaments, (Manel et al., 2003), into the co-culture reduced the percentage of Tax positive HUT78 cells, *i.e.* reduced HTLV-1 *de novo* infection, by 55.9%. However, cytochalasin B appeared to affect MT-2 cell viability (Supplementary Fig. 4A) and Tax expression levels (Supplementary Table 3) so a potentially detrimental effect of cytochalasin B on the infectivity of MT-2 cells reducing their infectiousness could not be ruled out. Treatment of MT-2 cells with sodium valproate, which inhibits histone deacetylation, had no effects on their viability (Supplementary Fig. 4A) nor Tax expression (Supplementary Table 3 & Supplementary Fig. 4B). In our second inhibition study, including sodium valproate in the co-culture reduced the resulting percentage of Tax expressing HUT78 cells, *i.e.* reduced HTLV-1 infection of HUT78 cells, by 23.5% (Fig. 3).

## 4 Discussion

We sought alternative methodology to examine early *de novo in vitro* HTLV-1 infection; the rationale being that the most commonly used current methodology employs DNA extraction from co-cultures reliant on irradiation induced donor cell death, and their subsequent removal over time, not suited for early infection studies. We recognised that small percentages of donor cells appear resistant to irradiation and maintain their viability in culture; DNA from irradiation resistant cells might contaminate DNA extractions from co-culture systems producing false-positive detections of HTLV-1 DNA by qPCR. We hereby present methodology to measure earlier ‘true’ *de novo in vitro* HTLV-1 infections *via* flow cytometric detection of induced Tax expression in newly infected cells.

After 24 h co-culture with HTLV-1 donor MT-2 cells >2% of permissive HUT78 cells, a male HTLV-1 negative cell line derived from a lymphoma patient with Sezary syndrome (Bunn and Foss, 1996), started to express detectable HTLV-1 specific Tax protein visualised by the highly specific anti-Tax antibody, clone Lt-4 (Tanaka et al., 1990), using flow cytometry. We conclude that this is *de novo* Tax expression in previously Tax negative cells indicative of DNA integration into the HUT78 genome and hence *de novo* HTLV-1 infection. We recognise the possibility of exosomal or syncytial transfer of Tax (Sun et al., 2006) as a small but potential caveat to our methodology. Detection of Tax expression was selected over that of p19 or gp46 due to potential methodological issues of membrane-transfer during co-culture and it would be interesting to examine *de novo* expression of any of these markers, and thus *de novo* infection, in cell-free infection studies that these methodological caveats would not affect. In our opinion this methodology is not limited to examining infection only of HUT78 cells by MT2 cells. This methodology would be transferable to study infection between cell types available to other researchers. MT2 and HUT78 were selected here only to establish this methodology, based upon a hypothesis that they were the most infective, based on higher expression of Tax than HUT102′s, and permissive, due to higher expression of neuropilin and GLUT-1 than CEM and Jurkat’s of the lines available to us.

An important methodological note is that several secondary antibodies were trialled and all but a highly specific, cross absorbed, anti-IgG3 secondary antibody recognised Lt-4 non-specifically (data not shown) (Goon et al., 2002). Similarly the anti-IgG3 antibody was carefully titrated since usage above 1:500 (0.2 μg/ml) produced non-specific binding (data not shown); antibody titration is likely to be required for each cell type and any alternative application used. Our secondary antibody, being SureLight APC conjugated, defined usage of the CellTracker orange dye; our methodology is adaptable if alternatively conjugated secondary antibodies are used with an appropriately segregating CellTracker dye, with several different fluorescent emitters available commercially.

Previously, Mosley et al utilised flow cytometry to show Tax expression in HTLV-1 infected cells but did not look at *de novo* infection (Mosley et al., 2006). Lt-4 antibody has been used to demonstrate HTLV-1 infection of Jurkat cells (Toulza et al., 2010) and cell-free virus infection of monocyte-derived dendritic cells (Manuel et al., 2009) and Rikzallah et al reported dendritic cell infection by measuring Lt-4 levels using flow cytometry (Rizkallah et al., 2017). However, to our knowledge, detection of Tax using Lt-4 has not been previously used to examine *de novo* HTLV-1 infection at early time points nor has this methodology been used to assess the ability of candidate drugs to inhibit infection. Our use of Lt-4 as a marker of HTLV-1 infection relies on efficient segregation of donors cells through flow cytometric gating. This could be achieved using forward and side scatter properties in flow cytometric analyses with the different properties of the two cell types in our system but we chose to use an additional cell identifier, a cell tracker dye in the permissive cells, to make the identification process more robust. A conservative gating strategy is notable to avoid detection of donor cells, with this being operator controllable in our system.

Our *de novo* infection could be effectively blocked by two known *in vitro* HTLV-1 infection inhibitors: cytochalasin B [13] and sodium valproate [14]. Inhibition levels were consistent with others and we believe our methodology could identify novel drugs in ligand screening studies of semi-high-throughput given our assay’s capability of determining early infection / infection-inhibition. Reporter cell systems have also been developed (Gross and Thoma-Kress, 2017) that offer a similarly effective alternative approach to qPCR methodologies to study HTLV-1 infection and have also been proposed to be useful to study novel infection inhibitors (Abdizadeh et al., 2017). However, we propose that our methodology has the advantage over reporter cell systems and therefore could also be utilised to study *de novo* HTLV-1 infection of cervical tissue in more physiologically relevant organo-typical models, reminiscent of the studies employed to study HIV-1 infection (Dezzutti et al., 2013). Preliminary studies not described here used MT-2 or C1866 (negative control) cell co-culture with *ex vivo* primary human cervical tissue followed by cervical tissue digestion using collagenase II (Hagman et al., 2012) and a gentleMACs dissociator available from Miltenyi Bioscience (Messier et al., 2012) to isolate single cells suitable for flow cytometry. Cervical cells were labelled with antibodies against p19 and gp46, as described here, and a small percentage of non-donor cells (CD3^+^/CD4^+^, non-MT-2, cervical T cells) were analysed with the finding that C1866 cells induced no meaningful expression of p19 and gp46 whilst substantial levels were found on cells from tissue co-cultured with MT-2 cells (data not shown). This was a promising and encouraging initial result but significant future optimisation would be required and expression of Tax should be optimised as the *de novo* infection marker. Using a methodology which detects early HTLV-1 *de novo* infection and its blockade reliably, is necessary for future pre- and post-exposure prophylaxis as well as microbicide drug testing.

## 5 Conclusions

Effective experimental techniques are required to detect *de novo* HTLV-1 infection *in vitro* at early time points; we now show that the detection of *de novo* HTLV-1 Tax expression in permissive cells is possible through the use of a highly selective anti-Tax antibody and a specific secondary antibody coupled with cell-tracker labelling and effective gating strategies to discriminate between newly infected- and donor-cells by flow cytometry. We believe this is a technological advance that provides an alternative to current methodologies, particularly at early time points of infection, and could contribute to increased understanding of HTLV-1 infection mechanisms and potentially clinically relevant inhibitor investigations.

## Author contributions

JF devised the study and designed the experiments, YT provided the antibodies, CP and JF performed the experiments and analysed the data. FM and YT provided intellectual input. JF and FM wrote the manuscript; all authors approved the final version.

## Acknowledgements and Funding

We acknowledge Graham Taylor and Charles Bangham, both from Imperial College, London, UK, for provision of MT-2 cells and input towards our secondary antibody optimisation, respectively, and David Brighty, University of Dundee for helpful discussions. This work was funded by a University of York Department of Biology summer studentship grant awarded to JF (M0225901), and part funded by The Wellcome Trust Value in People award to JF reference 092430/Z/10/Z and a University of York/Hull York Medical School pump-priming award to FM (M0227004).

## Declaration of Competing Interest

None.
