# Supplementary material for: Flow cytometric methodology for the detection of de novo human T-cell leukemia virus -1 infection in vitro: A tool to study novel infection inhibitors
Source: J Virol Methods. 2019 Dec;274:113728. doi: 10.1016/j.jviromet.2019.113728 (PMC6853161; doi:10.1016/j.jviromet.2019.113728)

Flow cytometric methodology for the detection of *de novo* human T-cell leukemia virus -1 infection *in vitro*: A tool to study novel infection inhibitors

Carina Peres, Yuetsu Tanaka, Fabiola Martin & James Fox*

*james.fox@york.ac.uk

Abstract

Methodology to detect and study *de novo* human T-cell leukemia virus (HTLV)-1 infection is required to further our knowledge of the viruses' mechanisms of infection and to study potential therapeutic interventions. Whilst methodology currently exists, utilisation of an anti-Tax antibody to detect *de novo* Tax expression in permissive cells labelled with cell tracker allowing for the detection by flow cytometry of new infection after co-culture with donor cell lines productively infected with HTLV-1 is an alternative strategy. Using this methodology, we have been able to detect *de novo* infection of the T cell line HUT78 following co-culture with the productively infected HTLV-1 donor cell line MT-2 and to confirm that infection can be effectively blocked with well characterised infection inhibitors. This methodology will benefit experimental studies examining HTLV infection *in vitro* and may aid identification of therapeutic agents that block this process.

Keywords: Tax, flow cytometry, HTLV-1, human T-lymphotropic virus-1, human T-cell leukemia virus

Supplementary Information

Index

		Pages
1. Supplementary Methodology		2-4
2. Supplementary Results	Supplementary Table 1	5
	Supplementary Table 2	5
	Supplementary Table 3	5
	Supplementary Figure 1	6
	Supplementary Figure 2	7
	Supplementary Figure 3	8
	Supplementary Figure 4	9

1. Supplementary Methodology

1.1 Cell culture

HUT78 and MT-2 cells were cultured as described in the manuscript. CEM (gift from Mark Coles, University of York) and C1866 (gift from Graham Taylor, Imperial College London, UK) cells were cultured in the same medium under the same conditions. Jurkat E6-1 (gift from Mark Coles, University of York), and HUT102 (ATCC / LGC standards, Teddington, UK) cells were cultured in the same medium but containing 1 mM sodium pyruvate (Sigma-Aldrich, Dorset, UK) and 4500 mg/l glucose (Sigma-Aldrich).

1.2 Irradiation and cell counting

Irradiation was defined as high (200 Gy) or low dose (30 Gy) and delivered to cells resuspended in growth media at $1x10^6$ cells/ml in 50 ml falcon tubes using an RS 2000 X-Ray generator containing a Comet MXR-165 X-Ray source; control aliquots of cells received the same handling but no irradiation. Following irradiation, cells were immediately (T0) counted using trypan blue and exclusion counts of live cells/ml were calculated. Cells were returned to culture flasks under standard tissue culture conditions for 10 days. Exclusion cell counts were performed every subsequent 24 hours with media replenishment at day 3 and 6, adjusting the cell concentration to $0.5x10^6$ cells/ml on both occasions.

1.3 Immunofluorescence

Glass coverslips (round, 13mm No. 1.5, Scientific Laboratory Supplies, Nottingham, UK) were poly-d-lysine coated (Sigma-Aldrich) and allowed to dry. 200,000 cells per coverslip were applied in 1 ml standard growth media and left to adhere overnight under standard tissue culture conditions. Medium was removed, cells were fixed with 3% PFA (Polysciences Europe GmbH, Hirschberg an der Bergstrasse, Germany) in PBS for 20 minutes at room temperature then free-aldehyde groups were quenched by a 20 minute incubation with 50 mM NH_4Cl in PBS. Permeabilisation buffer was prepared, containing 0.05 % saponin (Sigma-Aldrich), and coverslips were washed three times. Block buffer, permeabilisation buffer containing 1% FCS and 1% human IgG (Sigma-Aldrich), was then applied for 30 minutes. After this time the block buffer was removed and 5 µg/ml primary anti-Tax antibody, clone Lt-4 (kindly provided by Tanaka Y, Ryukyu University), or the same concentration of IgG3 isotype control (eBioscience) was applied in permeabilisation buffer containing 1% FCS (staining buffer). Antibody was incubated for 1 hour at room temperature before 3x 5 minute washes in permeabilisation buffer. Secondary antibody, goat anti-mouse IgG3, alexa

fluor 488 (Invitrogen, Paisley, UK), in staining buffer at 4 µg/ml was then applied for 1 hour at room temperature. 3x 5min washes with permeabilisation buffer then 2x washes in PBS before DAPI (Sigma-Aldrich) was applied at 1 µg/ml in PBS for 5 minutes. Coverslips were mounted on microscope slides in Mowiol (Sigma-Aldrich) and allowed to dry. Images were taken at the same settings on an Olympus BX51 microscope and MagnaFire SP software (Olympus, Southend-on-Sea, UK) before images were combined in Zen lite (Carl Zeiss Microscopy Ltd, Cambridge, UK).

1.4 Flow cytometric analysis

As described in the main text methodology but additionally here using optimised concentrations of p19 or gp46 (both from Y. Tanaka) and a titrated concentration of appropriate fluorescently labelled secondary antibody.

1.5 Effect of sodium valproate or cytochalasin B on MT-2 cell viability and Tax expression

MT-2 cells were cultured in the presence of 10 µM cytochalasin B (Fisher Scientific, Loughborough, UK) or 0.5 mM sodium valproate (Santa Cruz Biotechnology, Heidelberg, Germany) or left untreated but with the addition of an equivalent volume of vehicle control. At stated time points, differential live / dead cell counts were made utilising trypan blue cell exclusion and DNA was extracted from aliquots of cells for *TAX* quantification by real-time PCR.

2. Supplementary Results

Supplementary Table 1. Representative relative expression levels (mean fluorescence intensity after isotype subtraction) of neuropilin and GLUT-1 on three different T cell clonal lines.

Cell line	Neuropilin expression	GLUT-1 expression
HUT78	268	1046
Jurkat	70	405
CEM	55	35

Supplementary Table 2. Representative relative expression levels (mean fluorescence intensity after isotype subtraction) of p19, gp46 and Tax on HTLV-1 chronically infected cell lines.

Cell line	p19 expression	gp46 expression	Tax expression
MT-2	2008	1462	84836
HUT102	986	240	4937

Supplementary Table 3. Average Tax expression levels (mean fluorescence intensity after isotype subtraction) in MT-2 cells untreated or after treatment with sodium valproate or cytochalasin B.

	Untreated	+ sodium valproate	+ cytochalasin B
Tax expression	19,518	21,219	14,774

Supplementary Figure 1: MT-2 cells persist in culture up to 10 days after high or low dose exposure to X-ray irradiation. MT-2 cells were exposed to high (200 Gy) or low dose (30 Gy) X-ray irradiation or kept under the same conditions but not exposed to irradiation (Non-treated). Live cell counts were made immediately after treatment and every 24 hours after returning the cells to culture. Mean ±SEM cell counts from two independent experiments are displayed.

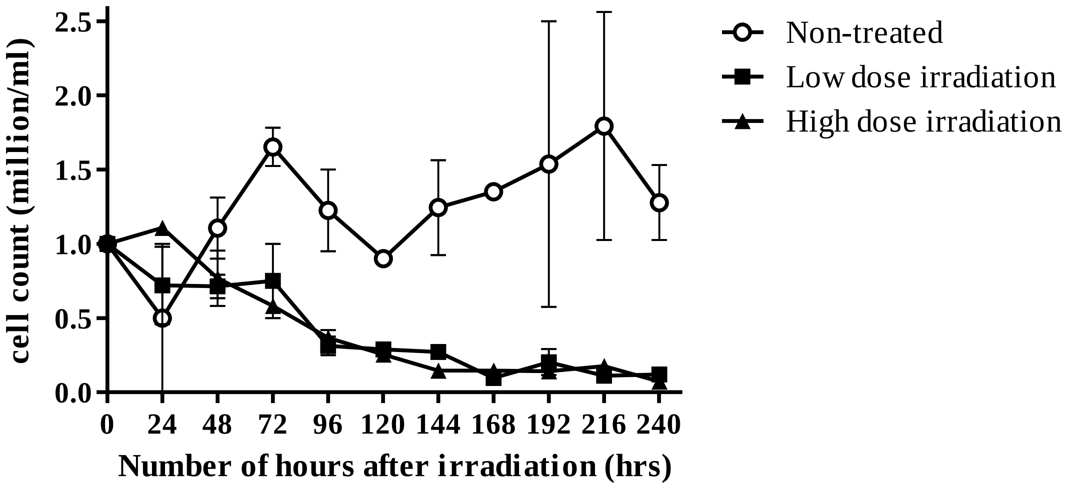

Supplementary Figure 2: HUT78 cells readily take up cell tracker dye (related to Figures 1 and 2). HUT78 cells (red events) non-stained (A) or stained with cell tracker (blue events in gate P3) (B) can be readily identified by fluorescence from MT-2 cells (green events in gate P2) (C). Only events in P3 are analysed for Tax expression levels in HUT-78 cells. Representative dot plots are shown, x axis represents fluorescence intensity.

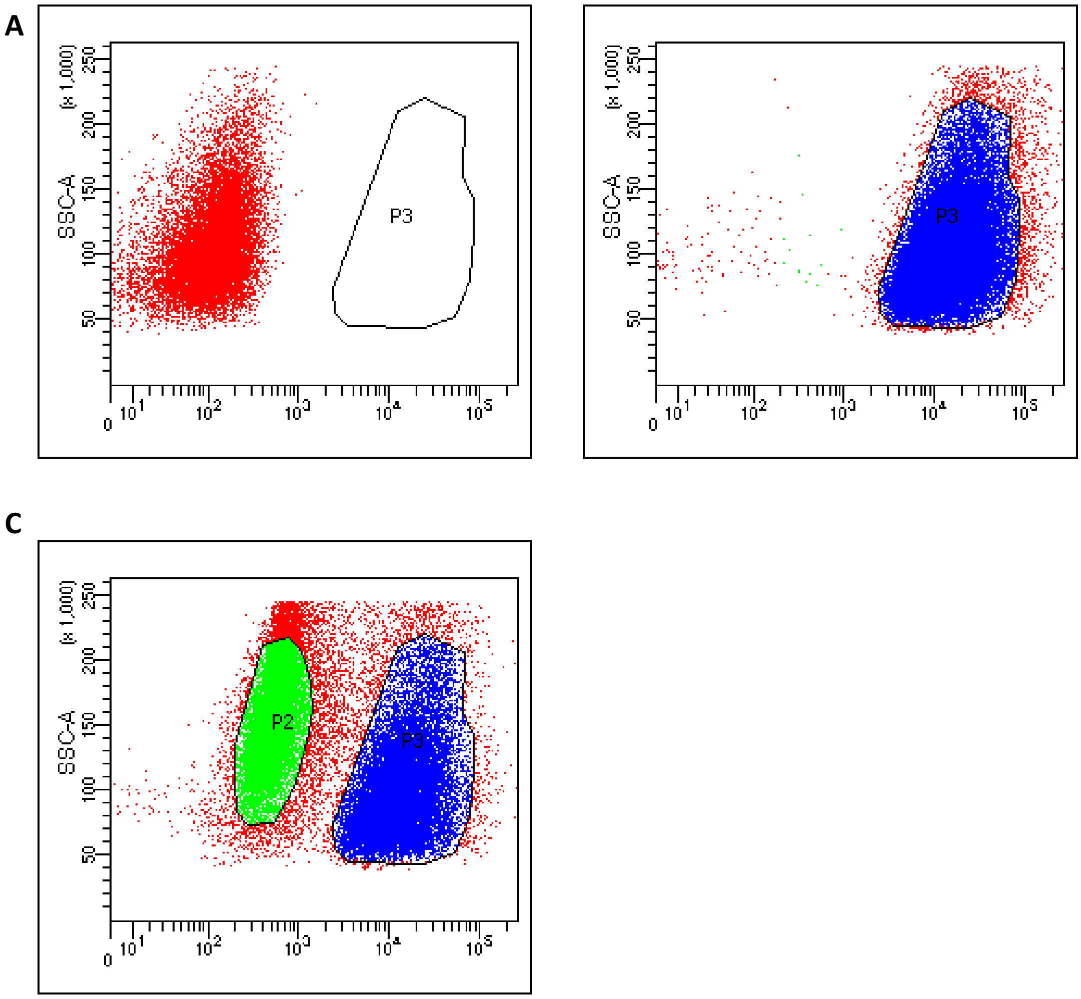

Supplementary Figure 3: HUT78 are Tax negative whilst MT-2 are positive (related to Figure 1). Representative immunofluorescent staining of HUT78 cells (A, B) or MT-2 cells (C, D) with IgG3 isotype control (A, C) or the anti-Tax antibody (B, D) followed by an anti-IgG3 secondary antibody coupled with alexa-488 (green). Images were acquired using the same conditions. Cells are counterstained with DAPI (blue).

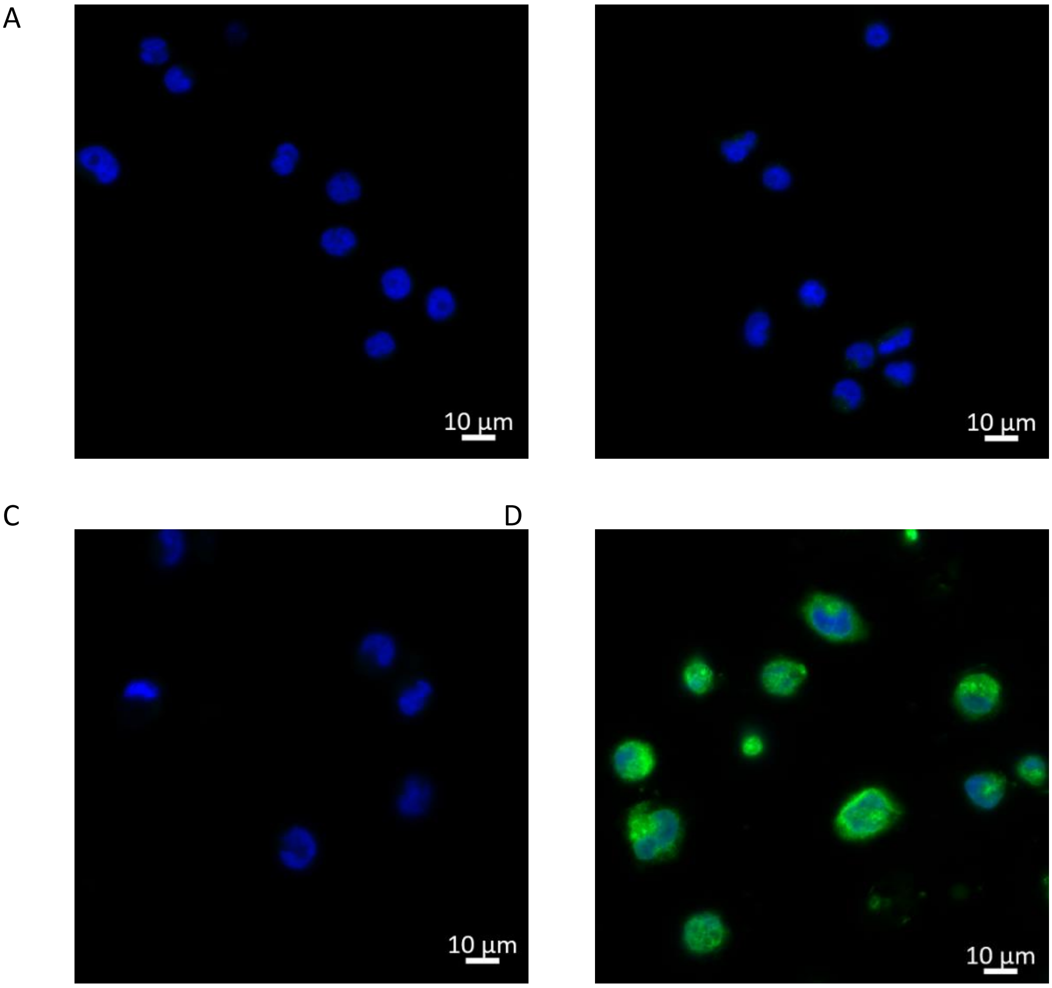

Supplementary Figure 4: Cytochalasin B and sodium valproate have no detrimental effect on Tax expression in MT-2 cells but cytochalasin adversely affects MT-2 cell viability (related to Figure 3). MT-2 cell viability (A) and Tax expression levels (B) were calculated at stated time points after treatment with 0.5 mM sodium valproate or 10 μM cytochalasin B. Data is a single representative experiment.

A

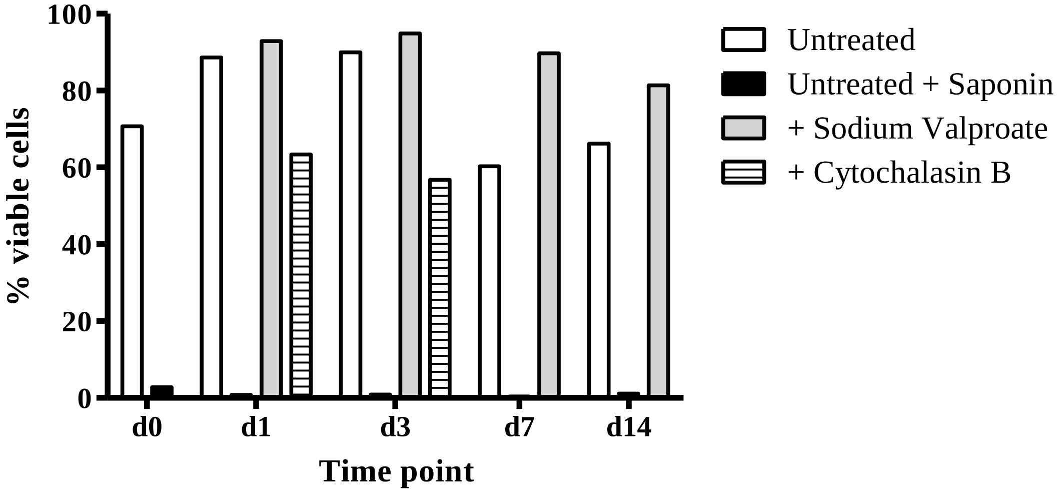

B

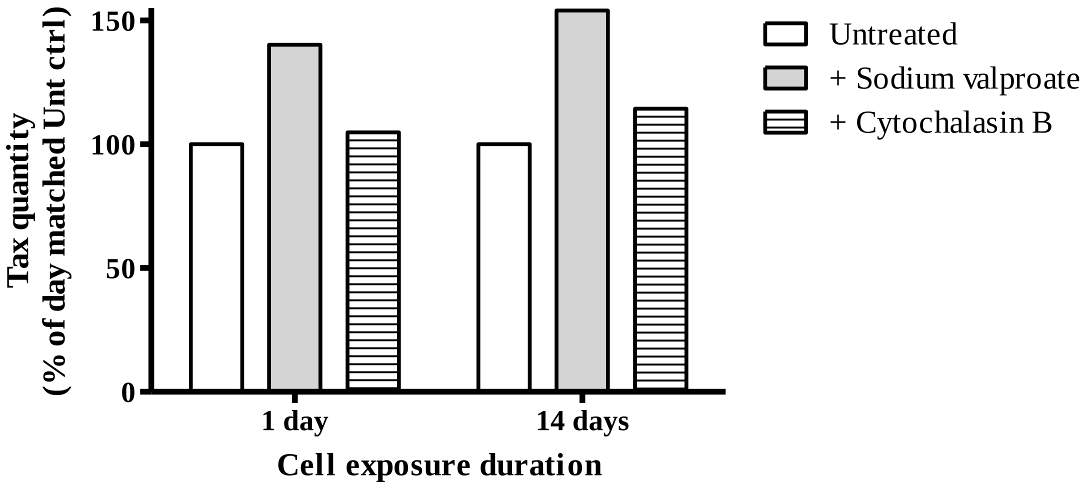

Supplement: Supplementary file 1 [file mmc1.pdf]
